# Non-invasive neuromodulation to improve gait in chronic multiple sclerosis: a randomized double blind controlled pilot trial

**DOI:** 10.1186/1743-0003-11-79

**Published:** 2014-05-01

**Authors:** Mitchell E Tyler, Kurt A Kaczmarek, Kathy L Rust, Alla M Subbotin, Kimberly L Skinner, Yuri P Danilov

**Affiliations:** 1 Department of Biomedical Engineering, University of Wisconsin, Madison, WI 53706, USA; 2 Department of Orthopedics and Rehabilitation, University of Wisconsin, Madison, WI 53706, USA

**Keywords:** Multiple sclerosis, Neuromodulation, Balance, Gait, Tongue, Neurostimulation, Non-invasive, Electrical stimulation

## Abstract

**Background:**

This study sought to examine the effect of targeted physical therapy with and without cranial nerve non-invasive neuromodulation (CN-NINM), on the walking ability of people with MS who exhibited a dysfunctional gait. We hypothesized that subjects who received electrical stimulation would have greater improvement than those who had a control device after a 14-week intervention. Gait disturbance is a common problem for people with multiple sclerosis (MS). Current management may include exercise, pharmacology, functional electrical stimulation, compensatory strategies, use of assistive devices, and implanted electrical devices. We have developed an effective rehabilitative strategy using neuromodulation of the cranial nerves via electrical stimulation of the tongue to enhance the plasticity of the brain.

**Methods:**

The study is a within-subject blinded randomized control design. Twenty chronic MS subjects with an identified gait disturbance were assigned to either an active or control group. Both groups completed a 14-week intervention program using a standardized combination of exercise and a device that provided electrical stimulation to the tongue. Those in the active group received electrical stimulation on the tongue that they could perceive. Those in the control group used a device that did not provide a physiologically significant stimulus and was not perceivable. Subjects were assessed with the Dynamic Gait Index (DGI).

**Results:**

The DGI scores improved for both groups. There were significant between-group differences, with the active group showing statistically greater improvement than the control group mean.

**Conclusion:**

People with MS demonstrated improved gait with CN-NINM training in a pilot randomized controlled trial. This study suggests that tongue-based neurostimulation may amplify the benefits of exercise for improving gait in people with chronic MS.

## Background

Individuals with moderate multiple sclerosis (MS) present with an array of symptoms, with walking impairment being among the most common. Gait speed, cadence, stride length and time spent on double-limb support are frequently affected, and correlate with reduced independence and productivity, impacting overall quality of life [1]. It is a major factor in estimates of disease progression [2-4], and approximately 40% of MS patients will need some form of walking assistance within 15 years of disease onset [5]. Consequently, interventions targeting gait disturbance in patients with MS are in demand. Currently these include rehabilitation therapy and pharmacological management.

While therapeutic exercise has long been believed to increase symptoms, more recent evidence has demonstrated that exercise and increased physical activity are beneficial for people with MS, and are becoming more commonly included as part of treatment interventions [6-10]. The optimal type or mode of exercise, intensity, frequency, duration and maintenance of exercise training to manage the progression of balance and gait dysfunction in MS has, however, yet to be established through randomized trials [3].

Rehabilitation therapy includes occupational, physical and speech therapies as part of the comprehensive management of symptoms [11,12]. Compensatory strategies such as energy conservation, use of adaptive equipment, and environmental adaptations are often utilized in these interventions [13-20]. Medical devices and physical modalities may also be used in multidisciplinary interventions [21]. A recent Cochrane review of multidisciplinary rehabilitation for adults with MS did not find strong evidence of interventions that resulted long-term improvements [12].

Pharmacological management may slow disease progress or reduce motor symptoms. For example, Ampyra (Acorda Therapeutics) was approved after it was shown to be effective in improving walking speed by 20% when compared to a placebo group that improved 8% [2]. This outcome, however, was observed in only 35% of those tested, and many participants reported significant side effects. Additionally, there is little evidence that these drugs will prevent the mobility disability of persons with 2^nd^ stage MS (Kurtzke Expanded Disability Status Scale (EDSS) score of 4.0 or greater) [1,3,22,23].

The medical devices and physical modalities utilized in treatment for people with MS vary. Functional electrical stimulation, as delivered through electrodes attached to the skin, has been shown to be effective in improving gait, as long as the electrodes are attached and the stimulation unit is on [24-29]. Other contemporary forms of neurostimulation (aimed at induced neuromodulation) are invasive, expensive and have the potential for adverse effects. For example, deep brain stimulation and vagus nerve stimulation, which use implanted pacemaker-like electrical devices, are indicated for decreasing tremors in MS, but carry surgical risks [30-33]. These therapies have not been widely attempted in MS rehabilitation. Neurostimulation that directly stimulates the peripheral or central nervous systems to improve motor impairments is still being investigated. Non-invasive neurostimulation that has been tried in people with MS are typically large clinic-based devices that employ powerful electromagnetic stimulation of the brain’s cortex (transcranial magnetic stimulation) [34,35], or electrodes that pass electric current through the skull (transcranial direct current brain stimulation) [36,37]. Intermittent theta burst stimulation of the motor cortex has been demonstrated to reduce spasticity in people with MS [38], and when combined with exercise therapy, to decrease fatigue [35]. Success with these interventions has, however, been limited. Consequently, there continues to be a need for alternative approaches that have the potential to help improve mobility in people with MS.

Previous studies have shown that the tongue can be used as an effective interface for sending electrical signals to the central nervous system [39-45], for example sensory substitution in balance-impaired or blind individuals [43,46-50]. Individuals with primary vestibular disorders who trained using electrical stimulation through the tongue coupled to head-position information demonstrated balance improvements that were sustained for weeks beyond the final stimulation session [46,51].

Using a different methodology that delivers tongue stimulation which, like the present study, is devoid of information linked to head position or any other exogenous variable, our recent functional MRI results indicated that the improvements that occurred with the neuromodulation training (using electrical stimulation on the tongue) are likely related to modulation of neural activity within structures of the brain that control balance and movement [44,45,52].

### Objective

This study sought to examine the effect of targeted physical therapy, with and without cranial nerve non-invasive neuromodulation (CN-NINM), on the walking ability of people with MS who exhibited a dysfunctional gait. We hypothesized that subjects who received electrical stimulation on the tongue would have greater improvement than those who had a control device after a 14-week intervention.

## Methods

### Study design

**Figure 1:**
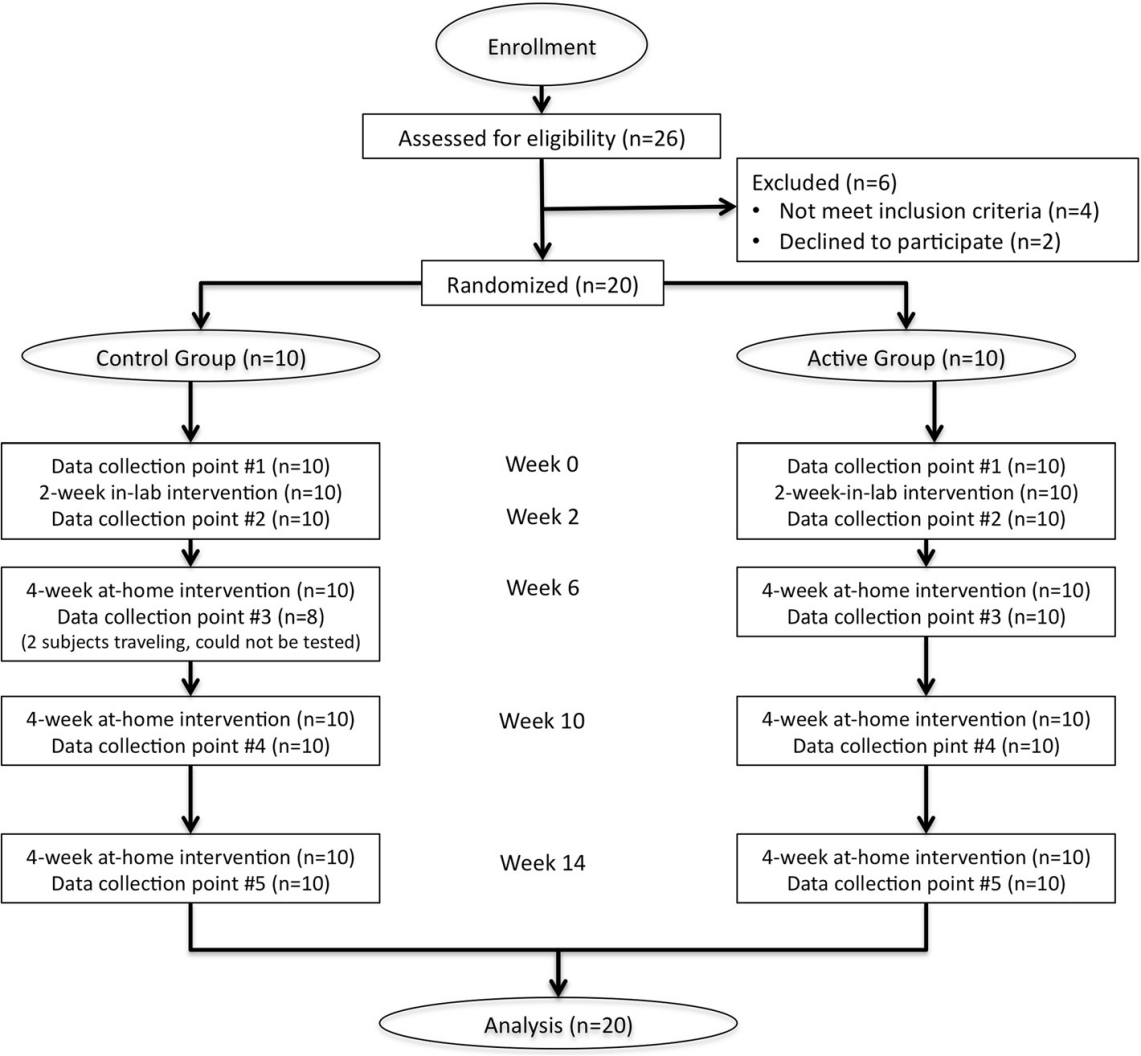
Detailed flow chart of the study intervention.

The study was a randomized, double blind controlled trial. Twenty subjects (males and females) with identified gait deficits due to the effects of MS were distributed into 2 treatment groups: 10 used an “Active” CN-NINM device, or Portable Neuromodulation Stimulator (PoNS™), and 10 used a “Control” (very low stimulation) device. The study consisted of two phases: 2 weeks of twice-daily gait training in the laboratory while using the PoNS, followed by 12 weeks of the same daily routine at home. All subjects performed the same gait training over the period of the study. All subjects were tested at the beginning and end of the first phase, and every 4 weeks during the second phase. The structure of the study is depicted in Figure 1.

### Subjects

Twenty-six subjects with stable symptoms from MS were recruited by physician and subject referrals. Because gait impairment can be manifested in persons with any type of MS, we thought it was important to include people with any type of MS who were affected by a walking impairment. Inclusion criteria were: relapsing remitting (RRMS), primary progressive (PPMS), or secondary progressive (SPMS) without relapse within 6 months of enrollment in the study; EDSS scores of 3.5 to 6.0; no changes in medication within 3 months of enrollment; and ability to walk 20 minutes on a treadmill (with handrail support as needed) without rest. The EDSS is a rating system used for classifying the condition of people with MS, and emphasizes walking ability. Scores in the range of 3.5 to 6.0 on the EDSS represent people who are ambulatory and have a few functional systems affected to those more significantly affected, requiring intermittent or constant unilateral assistance (e.g., a cane, crutch, or brace) to walk 100 meters with or without resting.

Exclusion criteria were: major co-morbidities, especially other neurological disorders, uncontrolled pain, hypertension, diabetes, or oral health problems. A summary of the subjects’ general characteristics is presented in Table 1. Three subjects (2 Control, 1 Active) were on neurostimulating medication (e.g. fampridine) that might alter motor function. Five subjects were on anti-inflammatory medications, six on antispasmodics, and six had non-narcotic prescriptions to manage chronic pain. Four candidates were not enrolled because they did not meet the inclusion criteria, and two did not participate because they could not meet the time commitments of the study.

**Table 1:** Subject characteristics at baseline

	**Active**		**Control**		
**Subjects**	**10**		**10**		
**Men/Women**	**4/6**		**2/8**		
	**Mean**	**SD**	**Mean**	**SD**	***p***
Age	55.40	8.73	51.90	9.31	0.40
Years with MS	24.10	11.03	13.10	6.72	0.01*
EDSS	5.25	0.98	4.60	1.05	0.17
DGI	8.90	2.85	11.95	4.04	0.07

*Indicates significant difference.

*EDSS*, Expanded Disability Status Scale, DGI = Dynamic Gait Index.

The University of Wisconsin-Madison Health Sciences Institutional Review Board approved this study and all subjects gave written consent before participation.

### Randomization and blinding

Subjects were randomly assigned to either the control or active group by the primary investigator (PI) as they enrolled in the study. Ten subjects were assigned to each group, with no regard given to age, gender, individual EDSS score, disease state, functional status, or chronicity of MS. The PI provided each subject with the proper neuromodulation device and instructed the subject in its use. The subjects in the active group used a device that provided electrical stimulation on the tongue that they could perceive. Those in the control group used a device that provided a stimulus that was not perceivable. Subjects were instructed that this was controlled study investigating the effects of stimulus level so they may or may not feel the stimulation. To avoid deception, all subjects in both groups were assured that they were in fact receiving stimulation, whether or not they could feel the stimulus. The subjects and the therapists providing the intervention and testing were not informed which group a subject was assigned. In order to maintaining blinding, both subjects and therapists were instructed to not discuss any details of the stimulus sensation with each other. Additionally, all subjects were instructed to not adjust the stimulus intensity in the presence of the therapist. All questions about device use or the stimulation were to be addressed only to the PI. A summary of subject distribution in the two cohorts is presented in Table 2.

**Table 2:** Multiple sclerosis subgroups

	**Active (10)**	**Control (10)**
Relapsing Remitting	5	8
Male/Female	1/4	2/6
Secondary Progressive	5	1
Male/Female	3/2	0/1
Primary Progressive	0	1
Male/Female	0/0	0/1

### Experimental procedures

#### Intervention

The goal of the training was to develop a more normal gait pattern. The twice-daily intervention was identical for each subject, and progressed in two phases: a 2-week twice daily in-lab phase, followed by a 12-week at-home phase where subjects performed the same training as instructed in the laboratory. The structure and progression of both the in-lab and at-home interventions, as well as the assessments, are shown in Figure 1. Subjects were telephoned weekly during the at-home phase to ensure compliance with the protocol. In order to maximize any potential benefit from participation, the degree of challenge in each subject’s training program was increased according to the progress they made at each 4-week follow-up visit for testing and retraining.

#### Device

**Figure 2:**
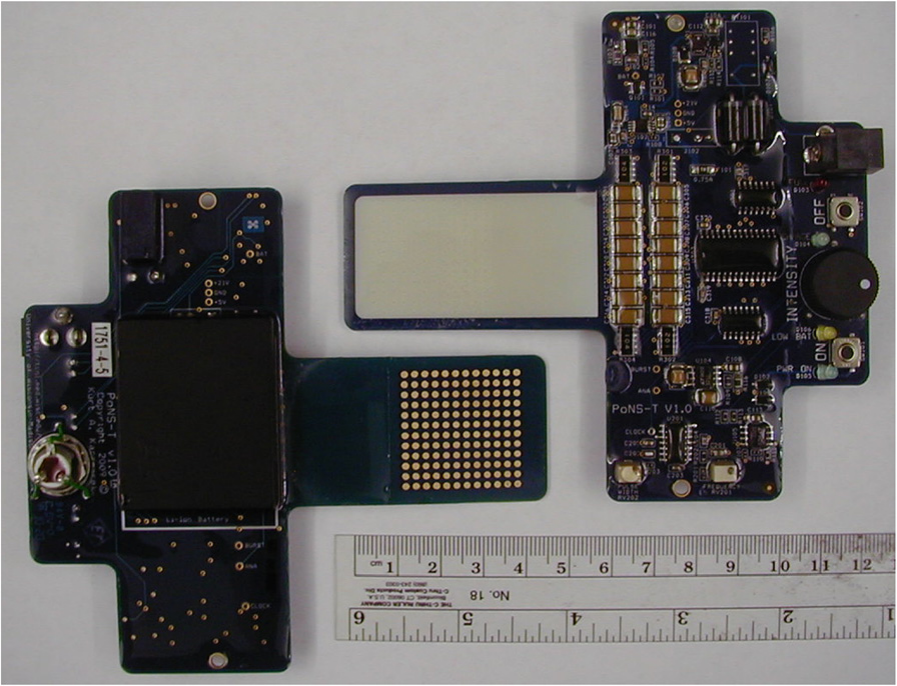
Portable Neuromodulation Stimulator (PoNS™) device, top and bottom view.

Electrical stimulation to the tongue was delivered via the Portable Neuromodulation Stimulator (PoNS™) device shown in Figure 2. The PoNS™ device was held in place lightly by the lips and teeth around a rectangular tab that goes into the mouth and rests on the anterior, superior part of the tongue. The tab has 144 exposed gold-plated circular electrodes (1.5 mm diam., on 2.3-mm centers) on a 3 cm × 3 cm square matrix on a rigid printed circuit board that is coated with a biocompatible epoxy (Epotech 302-3 M, Epoxy Technology, Billerica, MA). The 12x12 array is divided into nine 4x4 sectors. Only one electrode in each sector is pulsed at any given time; the remaining electrodes serve as the return current path. The stimulation on each electrode is a triplet of 50 μs-wide positive pulses delivered at 200 pulses/s every 20 ms. Capacitive coupling ensures zero net direct current to minimize the possibility of tissue irritation. Electrode and waveform parameters were derived from earlier research aimed at developing electrotactile stimulation that is maximally comfortable and controllable [53-56], and implemented using circuitry similar to that in the earlier Tongue Display Unit [51].

Device function is user-controlled by buttons for ‘On’ and ‘Off,’ and subjects could adjust the stimulus level (pulse amplitude) from 0 to 17 volts by manipulating a knob on the device (see Figure 2). The “Control” version of the device was physically identical to the Active device, but delivered a stimulus at approximately 1/1,000 the minimum perceivable level. Here, adjustment of the intensity knob did not change this level.

To avoid bias or deception, subjects in both groups were provided with identical instructions to adjust the intensity level by turning the knob. They were told that they may or may not feel the stimulation. If they could feel the stimulus, they were to adjust the intensity until it was strong but not uncomfortable. We allowed individual adjustment rather than setting a fixed amplitude across all subjects, because of known inter-subject differences in tongue sensitivity, with the rationale that it is more important to hold invariant the result of the stimulation rather than its physical level [54]. From our previous research, we have observed that with experience, subjects set the intensity level at between 50 and 80% of the maximum dynamic range of sensation [43-48,50-52]. If subjects could not feel it, they were reassured that they were still receiving stimulation but it was below their individual threshold of perception.

#### CN-NINM training

Instruction and implementation of the 14-week intervention was identical for both groups. Subjects trained in the lab for 5 consecutive days (Monday through Friday) for 2 consecutive weeks working one-on-one with a therapist researcher for all in-laboratory training sessions. Subjects then continued the same training independently at home for the next 12 weeks. Subjects returned to the lab every 4 weeks for assessment, training review, and exercise program progression. Compliance was monitored daily in the lab, and weekly via self-reports by phone when the subjects trained at home.

The in-lab training consisted of two appointments per day. Within each appointment, subjects performed movement isolation exercises without the device, 20 minutes of gait training with the device, 20 minutes of balance training with the device, and 20 minutes of relaxation training with the device. The training was targeted to the specific ability of each individual, and they were provided with rest periods as needed. Subjects who were unable to complete the training components in the lab due to fatigue were allowed to delay that component until later in the day after they had had an opportunity to rest. Subjects were also expected to incorporate an additional relaxation session with the device at home each evening one hour before bed.

Subjects were instructed in movement isolation exercises (without device) at the beginning of each training session. These were geared to the ability of that individual. These exercises were designed to change abnormal movement patterns and re-train movements for improved neuromuscular control and mobility. Sample exercises were chin circles, shoulder circles, and hip circles. The emphasis was placed on quality of movements, not speed, and mirrors were used to provide visual feedback as needed. As the individual demonstrated competency with an exercise, new exercises were introduced.

During gait training subjects walked on a treadmill at progressive speeds and challenges designed to re-establish appropriate dynamic balance and gait patterns. Particular attention was given to symmetry of the gait pattern, including dynamic weight transfer, stride length, kinematics of the hip, knee, and ankle flexions/extensions, and bilateral symmetry of the stance and swing phases. In-lab gait training was performed on the treadmill for the first week, and both over ground and on the treadmill during the second week. Subjects were required to use a treadmill for at least 50% of the at-home training phase to control and monitor their training progress.

Gait training sessions were 20 minutes in overall duration. The first 5 minutes were performed at a comfortable pace. In the next two 5-minute periods the challenge was increased by changing a gait variable. The last 5-minute period was performed at a comfortable intensity but greater than that for the first 5-minute period. The variables were:

• Speed: from very slow to fast walking, focusing on maintaining gait kinematics without deterioration of performance;

• Grade: an incline increases effort and affects the relative involvement of the ankle, knee and hip joints;

• Support: use of handrails provides greater stability, but prevents arm swing and full weight transfer during the gait cycle. Challenge was created by decreasing hand contact time and force on the rails, with the goal of achieving arm swing commensurate with normal gait.

The therapist worked with the subject to determine an appropriate starting point relative to the subject’s baseline. The objective of each session of gait training was to start at a higher level than the previous session. The therapist used verbal and tactile cues as needed to correct posture and abnormal movement patterns during gait.

Balance training (with device) was performed by having the subjects stand on the floor or on foam with eyes closed, depending on their ability. As in gait training, balance training was targeted to each subject, advancing their challenge as they improved. To increase the balance challenge, subjects could change their stance width, foot position, or stand barefoot. Subjects stood close to a table for support if needed, and were guarded against falls by the therapist providing stand-by assist if needed.

Relaxation training (with device) was performed in an unsupported sitting position while wearing headphones and listening to theta-wave based sound tracks. Subjects were instructed in diaphragmatic breathing and maintaining relaxed attention.

### Assessments

Subjects completed the Dynamic Gait Index (DGI), a clinician-scored index of 8 gait tasks: normal walking, changing speed while walking, head turns and up/down tilts while walking, turning and stopping, walking around and stepping over objects, and traversing stairs [13,57-59]. This test was performed at baseline, after the 2-week in-lab intervention, and after 4, 8 and 12 weeks of at-home training (a total of 14 weeks intervention) for a total of 5 assessment points. Subjects were also instructed to provide daily (in-lab) and weekly (at-home) written documentation of their at-home training program in order to monitor compliance with the protocol.

The EDSS is a clinical tool for assessing and comparing patients’ global neurological disability and was not used to assess any changes in gait. It was used only for entrance criteria for the study. The final score reflects the status of many functional systems and may not be impacted by changes in gait. It has been shown to lack responsiveness to change and is therefore not recommended for research as an assessment tool for measuring change.

### Data analysis

Statistical analyses were completed with Systat version 8.0 (SPSS, Inc.). The demographic data presented in Table 1 were examined by descriptive statistics and the differences between active and control groups were compared using unpaired, two-tailed t-tests. DGI data summarized in Table 3 were also examined with descriptive statistics. DGI differences from baseline (week 0) were calculated for the 2, 6, 10, and 14-week test points and subjected to analysis of variance, separately for active and control groups; multiple comparisons for these analyses were performed with a Tukey HSD test and are shown in Table 3. Finally, the DGI differences from baseline were compared for the active and control groups using unpaired, two-tailed t-tests, separately for the 2, 6, 10, and 14 week data points. Although the DGI scale is technically categorical, it has been shown to have good psychometric properties [13,58-60]. As a precaution we repeated the latter analyses using the non-parametric Wilcoxon Rank Sum Test.

**Table 3:** DGI Mean Scores

	**Active**				**Control**			
**Week**	**N**	**Mean (SD)**	**Diff**^**a**^	***p***^**a**^	**N**	**Mean (SD)**	**Diff**^**a**^	**p**^**a**^
0	10	8.90 (2.85)			10	11.95 (4.04)		
2	10	13.30 (3.92)	4.40^c^	0.056	10	14.95 (4.29)	3.00	0.610
6	10	15.05 (3.53)	6.15^c^	0.003^b^	8	15.63 (4.73)	3.68	0.471
10	10	16.60 (3.95)	7.70^c^	<0.001^b^	10	16.75 (5.20)	4.80^c^	0.166
14	10	16.85 (3.40)	7.95^c^	<0.001^b^	10	15.40 (5.03)	3.45	0.745

^a^Difference from baseline (week 0).

^b^Statistically significant difference (*p < .05).*

^c^Clinically significant difference (> = 4).

## Results

Twenty people with MS participated in this study. The subjects were randomized into active and control groups as they enrolled. The groups were similar across age and EDSS scores (see Table 1). There was a difference between the groups for mean number of years with a diagnosis of MS (*p* = 0.01) and for baseline DGI, although the latter did not reach statistical significance. All subjects completed the 14-week intervention and the 5 data collection points except for 2 subjects in the control group who were unavailable for data collection point #3 due to travel.

**Figure 3:**
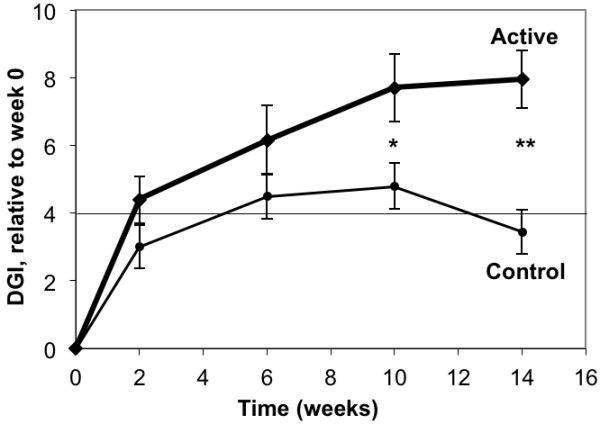
**Plot of Change in DGI score versus time within the study period.** The horizontal axis represents data acquisition time: 0 weeks is study entry (baseline), 2 weeks is end of lab training, and 6, 10, and 14 weeks are end of each 4-week home training period. The vertical axis represents the change in DGI relative to the baseline DGI value. Error bars are ± 1 SE; * indicates *p <* 0.05, ** indicates *p <* .005.

Subjects in the Active group achieved both statistically significant (*p* < 0.05) and clinically significant (DGI change of at least 4 points [13]) improvements in gait by the 6-week test point and these improvements continued through the 14-week test point (Figure 3 and Table 3). Subjects in the control group did not achieve a statistically-significant improvement in gait at any test points, although the apparent improvement at Week 10 would be considered clinically significant.

When the pairwise differences in DGI change from baseline (Table 3) are compared for the two groups, the active group shows a statistically-significant greater improvement than the control group at 10 weeks (*p* = 0.027) and 14 weeks (*p* < 0.001), using the independent t-test. (The Corresponding *p* values for the Wilcoxon test were 0.034 and 0.002).

The raw DGI data from this study are shown in Table 4^a^. It can be seen that every Active group subject (100%), and all but one in the Control group (90%) exhibited some improvement in their score between baseline and the end of the study at 14-weeks. The major difference is the magnitude of both the individual and mean group change between the two treatment groups. On average, the Active group improved by 7.95 points, while the Control group exhibited a mean change of 3.45 points.

**Table 4:** Raw DGI scores

**Group**	**Subject**	**DGI**	**DGI**	**DGI**	**DGI**	**DGI**
		**Baseline**	**After 2 weeks**	**After 6 weeks**	**After 10 weeks**	**After 14 weeks**
Active	1	8	8.5	9.5	9.5	12
Active	2	10	13.5	13.5	16	16.5
Active	3	10	16	18	17	20
Active	4	10.5	14.5	15.5	17.5	17
Active	5	6	10	12.5	13	14
Active	6	5.5	10	12	13	11.5
Active	7	7.5	10	14	17	17.5
Active	8	13	21	20	21	19.5
Active	9	13	17	16.5	22.5	21.5
Active	10	5.5	12.5	19	19.5	19
Control	1	11.5	16.5	18.5	19.5	15.5
Control	2	17	22		21.5	19
Control	3	13.5	16		18	16.5
Control	4	14	15	18	18	21
Control	5	14.5	14	16	17	16.5
Control	6	4	8.5	8	6.5	4
Control	7	15	20.5	20.5	21	18
Control	8	6	9	8.5	8	9.5
Control	9	12	15	18.5	18.5	18.5
Control	10	12	13	17	19.5	15.5

All subjects reported an increase in salivation at the outset of the study due to the presence of the device in the mouth. With instruction, each was able to develop swallowing strategies to manage saliva volume while maintaining the device in their mouth. Five subjects also reported mild headaches and temporo-mandibular joint pain during the first few days of participation. These symptoms were mitigated by the end of the 2-week in-lab training period by instructing them to not bite the array, relax their jaw, press the array firmly with the tongue, and breathe uniformly. Two RRMS subjects in the Active group experienced relapses requiring suspension of their participation, one for a period of 5 weeks, the others for 3 months. When they were able to resume training at the same level they had achieved prior to the relapse they were reintegrated into the study without adverse effect. Three subjects (2 Control, one Active group) experienced minor illnesses that required suspension of training for approximately 2 weeks each. Each was able to resume training without complication, and their testing schedules adjusted accordingly. Finally, several subject experienced fatigue after completion of the morning training session. They were encouraged to practice relaxation exercises to help them prepare for performing the afternoon sessions. This proved to be a useful skill for them to develop in managing their energy levels so that they could consistently complete their daily participation in the study.

## Discussion

This study investigated whether individuals with multiple sclerosis could improve their gait using CN-NINM intervention, a training program that incorporates exercises combined with noninvasive electrical stimulation of the tongue. The results show that subjects in the active group had greater improvements in their gait relative to the control group, and the improvements were clinically and statistically significant.

The sample size of this study was small (10 subjects per group), which may limit the external validity of the results. The sample size estimation for this study was based on the robust results of our earlier non-controlled proof of concept studies.

In considering sources of the induced improvement, our previous studies have demonstrated through fMRI that neuromodulation training using electrical stimulation of the tongue, combined with movement exercises in balance-impaired individuals, induces activity of the cerebellum and brainstem nuclei, structures of the brain that process balance and movement [44,45,52]. Additionally, Mori, et al. showed that using transcranial magnetic stimulation combined with exercise therapy resulted in a reduction in spasticity and fatigue [35]. These findings, combined with known afferent neural pathways from the tongue, suggest that tongue stimulation preferentially predisposes certain cerebellum and brainstem nuclei to beneficial neuroplastic effects resulting from movement exercises dependent on these nuclei, with the end result of improved movement control.

We hypothesize that CN-NINM induces neuroplasticity by noninvasive stimulation of two major cranial nerves: trigeminal, CN-V, and facial, CN-VII. This stimulation excites a flow of action potentials (AP’s) to the brainstem (pons varolli and medulla) and cerebellum via the lingual branch of the cranial nerve (CN-Vc), and chorda tympani branch of CN-VII. This effect of the stimulation extends to the corresponding nuclei of the brainstem – at least in the sensory and spinal nuclei of trigeminal nuclear complex and the caudal part of the nucleus tractus solitarius [44,45,52]. We postulate that the intensive activation of these structures initiates a sequential cascade of changes in neighboring and/or connected nuclei by direct collateral connections, brainstem interneuron circuitry and/or passive transmission of biochemical compounds in the intercellular space. There is evidence from related research that has observed changes in both neurotransmitter and other neuroactive compounds in response to chronic stimulation. Each AP in the trigeminal nuclei and brainstem releases up to 23 biologically active compounds including neurotransmitters that affect synaptic transmission, and the brainstem has the highest glia-to-neuron density anywhere in the CNS (50:1) [61]. The full function of the glial network is unknown, although it is generally agreed that it plays a vital role in the regulation of neural behavior through management of the chemical environment at the synaptic gap. Consequently, we believe the stimulation directly activates not only the neuronal network by electrical impulses (AP’s) but also the glial network by neurochemical impact. The net effect of this upregulation of neuroactive compounds is to potentiate the networks involved within the CNS and set the stage for sustained focal and global changes in brain behavior [62-68].

Improvement is also common in patients receiving a new intervention with a therapist [7,69]. A better indication of efficacy is if the patient improves when independently training at home. Here we see that both groups continued to improve during the at-home phase of the study. In a related study, Di Fabio, et al., found that patients with progressive MS who continued with an extended home outpatient rehabilitation over the course of 1 year experienced a lower rate of decline in physical function when compared with subjects who did not exercise [70,71]. Our study did not compare subjects who had performed the extended exercise protocol with any who had exercised and stopped. Consequently, to investigate this phenomenon, the natural progression of this study would be to repeat it using a longer intervention period.

In our study, though all subjects appeared to demonstrate improvements initially, only the active group continued to improve over the length of the study. It is likely that the early improvement in both groups was due to the intense involvement of the subjects with the trainers for the first 2 weeks of the study. It can been seen in Table 3 that improvements in performance for the active group continued to accumulate as subjects trained at home after the initial 2-week training phase. Subjects who trained using exercise only without stimulation (control group) continued to improve for the first month at home and then exhibited a plateau or even a decrease in performance. This provides preliminary evidence that the intervention is effective when performed independently at home.

We observed a significant difference between groups for number of years with MS, with those in the active group having had MS for a longer period of time. Because the progression of the disease varies from person to person, however, we did not feel that chronicity (years with MS) was as important as symptom presentation. We based our inclusion criteria on symptom presentation, specifically gait dysfunction, and EDSS score. We also observed that the mean initial (baseline) DGI score was lower in the active group (although this did not approach statistical significance), raising the concern that this may bias the results. Because of this apparent difference, we chose to analyze data using a simple DGI difference from baseline, rather than a % change as suggested by other groups which would have unduly favored the active group [60]. Similar analysis of % DGI change yielded identical conclusions.

We did not investigate whether the subtype of MS had an effect on the reported outcomes. It is acknowledged that because subject assignment was purely random there is some possibility that the disease subtype could affect sensitivity to the intervention and therefore the results. We note, however, that these sub-classifications relate primarily to the progression of the disease and not to their particular state during participation in the study. Furthermore, we excluded all candidates that had any changes in symptoms or medication in the 3 months prior to participation to ensure that their presentation was as stable as possible. Nonetheless, it would be beneficial to repeat this study using only MS patients of one particular type, such as PPMS or SPMS to determine if the rate or magnitude of change in performance differs as a function of the disease sub-classification.

The remaining question that may be posed is whether CN-NINM training could be practically deployed in a rehabilitation setting. The in-lab training for this study was admittedly time intensive (approximately 2 to 3 hours per day, per subject), involving far more time for therapy than a typical clinical setting would allow. This was an intentional departure from most therapeutic models for rehabilitation, derived from our prior experience with treating vestibular disorders [43,46]. Given the rigors of the intervention, and the nature of this neurodegenerative disease, this intensive in-lab phase was designed to ensure that subjects understood and could reliably perform the training program before using the device at home. The results suggest that this new paradigm for home-health rehabilitation, particularly for disorders previously deemed untreatable, is efficacious. Additional studies are necessary to determine if an abbreviated intervention would be as effective as the model presented here.

## Conclusions

The results of this pilot RCT demonstrate that non-invasive electrotactile stimulation, when combined with targeted physical therapy exercises, can significantly reduce clinical symptoms of gait dysfunction in multiple sclerosis. This complements a growing body of evidence demonstrating that neuromodulation combining electrical stimulation with exercise therapy has the potential to have a positive effect on motor control for people with neurological conditions. The results also demonstrate that the CN-NINM intervention shows promise for development as a clinical tool for improvement in gait in people with MS. Additionally, because the tongue stimulation device is portable it allows people to train at home, affording an efficacious therapeutic model not previously reported. This leads us to speculate whether people with other types of neurological conditions would benefit from this type of intervention to improve motor control. We suggest that further studies are warranted to investigate the breadth of applicability that this form of therapeutic intervention may have for meaningful neurorehabilitation.

### Endnotes

^a^While not formally approved, half-point scores (0.5) of the DGI are commonly used. The author of the DGI (Shumway-Cook) has acknowledged the limitations of the scale as originally constructed, and conceded that, if employed consistently by the clinician, the use of 0.5 interval is an acceptable interpolation of the defined condition on conventional scale.

## Abbreviations

(ANOVA): Analysis of variance; (CN-NINM): Cranial nerve non-invasive neuromodulation; (DGI): Dynamic gait index; (DGIB): Dynamic gait index baseline; (EDSS): Expanded disability status scale; (fMRI): Functional magnetic resonance imaging; (MA): Massachusetts; (MS): Multiple sclerosis; (RRMS): Relapsing remitting multiple sclerosis; (PoNS™): Portable neuromodulation stimulator; (PPMS): Primary progressive multiple sclerosis; (SPMS): Secondary progressive.

## Competing interests

Danilov, Kaczmarek, and Tyler have a financial interest in Advanced NeuroRehabilitation, LLC and in NeuroHabilitation Corp., which both have intellectual property rights in the field of use reported in this article.

## Authors’ contributions

MT, YD, KK, KR, AS, KS. 1 Study design. 2 Draft manuscript. 3 Statistical analysis. 4 Implementation of the intervention/study protocol. 5 Critical revision of manuscript. All authors read and approved the final manuscript.

## Authors’ information

All authors are affiliated with the University of Wisconsin, Madison.

MT teaches in the Department of Biomedical Engineering at the University of Wisconsin, Madison. He is a co-inventor of the PoNS device and is a contributor to the development of the CN-NINM training method. YD is a co-inventor of the PoNS device and is a major contributor to the development of the CN-NINM training method. KK is an expert in electrotactile stimulation and a co-inventor of the PoNS device. KR is an Occupational Therapist with an interest in human subjects rehabilitation research. AS is a psychiatrist who specializes in cognitive function and emotional processing. KS is a Physical Therapist with an interest in the application of neuromodulation for rehabilitation and is a contributor to the development of the CN-NINM training method.
